# Optimized point dose measurement: An effective tool for QA in intensity-modulated radiotherapy

**DOI:** 10.4103/0971-6203.37480

**Published:** 2007

**Authors:** Alok Kumar, Gautam Mukherjee, Girigesh Yadav, Vinod Pandey, Kalyan Bhattacharya

**Affiliations:** Advanced Medicare and Research Institute Ltd., Kolkata, West Bengal, India

**Keywords:** Beam profile, electronic equilibrium, intensity-modulated radiotherapy

## Abstract

In some cases of Intensity-modulated radiotherapy (IMRT) point dose measurement, there exists significant deviation between calculated and measured dose at isocenter, sometimes greater than ±3%. This may be because IMRT fields generate complex profiles at the reference point. The deviation arises due to lack of lateral electronic equilibrium for small fields, and other factors such as leakage and scatter contribution. Measurements were done using 0.125-cc ion chamber and Universal IMRT phantom (both from PTW-Freiburg). The aim is to find a suitable point of measurement for the chamber to avoid discrepancy between calculated and measured dose. Various beam profiles were generated in the plane of the chamber for each field by implementing patient plan on the IMRT phantom. The profiles show that for the fields which are showing deviation, the ion chamber lies in the steep-gradient region. To rectify the problem, the TPS (Treatment Planning System) calculated dose is found out at various points in the measurement plane of the chamber at isocenter. The necessary displacement to the chamber, as noted from the TPS, was given to obtain the optimum result. Twenty cases were studied for optimization, whose percentage deviation was more than ±3%. The results were well within tolerance criteria of ±3% after optimization. The mean percentage deviation value for the 20 cases studied, with standard deviation of 2.33 under 95% confidence interval, was found out to be 2.10% ± 1.14. Those cases that have significant variation even after optimization are further studied with film dosimetry.

Intensity-Modulated Radiotherapy (IMRT) is an advanced form of treatment compared to Three-Dimensional Conformal Radiotherapy (3DCRT). It is the application of varying-intensity beams along various target volumes in a rather complex way. Delivery of intensity-modulated fields is based on the use of computer-controlled multileaf collimators attached to modern linear accelerators. Since the beams are modulated in a rather complex way, each IMRT field often includes many small, irregular, off-axis fields resulting in isodose distributions for each IMRT plan that are more conformal to the tumor target volume than those from conventional treatment plans. This necessitates implementation of vigorous Quality Assurance (QA) practice, which includes machine- and patient-specific QA. The latter generally involves mapping the plan fields onto a phantom that has been computed tomography (CT) scanned, creating what is known as ‘Hybrid-Plan,’ and comparing the results with measurements made on that phantom. It is assumed that the validity of the results for the phantom can be extrapolated to the patient.[1]

When prostate tumors are treated with IMRT techniques, sparing of the rectum and bladder is a priority concern, together with adequate coverage of the Planning Target Volume (PTV). The close proximity of the prostate to the bladder and rectum often requires high-dose gradients in the interface regions, which result in highly inhomogeneous field fluences in the treatment plan. Ionization chambers are the preferred dosimeters for measuring absolute absorbed dose in IMRT fields.[2] Dose measurement with ionization chambers reflects the average dose value over their volumes.[3] Points at low-dose gradients are usually preferred for measurement purposes.[4] Escude L *et al.* had developed an optimization algorithm to find the most favorable points to position an ionization chamber for QA dose measurements of prostate cancer patients. The dose measurement was made in a plastic phantom at 287 optimized points.[5] Although other devices like multi-detector arrays, films and electronic portal imaging devices can be used, they are more suited to relative dose measurement. Also, Sanchez-Doblado F *et al.* had found that the absolute dosimetry in the penumbra region of the IMRT beamlet could suffer from significant errors. They have observed that the largest dose errors correspond to the smaller contribution of the corresponding IMRT beamlets to the total dose delivered in the ionization chamber within PTV.[6] Our present work is a parallel work based on the above studies. The aim of this study is to measure dose at a reference point in the phantom for IMRT treatment and to find out an optimized point of measurement in order to overcome large variation between calculated and measured dose without the help of another computer-generated algorithm.

## Materials and Methods

Twenty cases were studied for optimization, whose percentage deviation between TPS-calculated and ‘ion chamber’-measured dose was more than ±3%. These cases include head and neck, thorax and pelvic regions.

**Figure 1:**
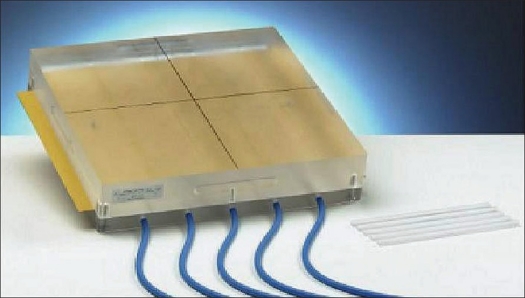
Universal IMRT phantom (courtesy PTW-Freiburg)

1. Linear accelerators: We have Elekta digital linear accelerators with 6 and 15 MV photon beams, fitted with a multileaf collimator having 40 pairs of leaves, each leaf having 1 cm width at isocenter, and 6 MV ‘step and shoot’ IMRT.
2. Virtual simulation: Oncentra virtual simulation software from Nucletron.
3. Treatment Planning System (TPS): Plato Sunrise 3D Treatment Planning System (TPS) with Inverse Treatment Planning (ITP).
4. IMRT Patient Plan: A custom immobilization device was fabricated, and computed tomography (CT) scan for each patient with 2-mm slice thickness was obtained. The Planning Target Volume (PTV) and Organ at Risk (OR) was delineated in Oncentra virtual simulation software. For the IMRT plan, five equally spaced and non-opposing beams were found suitable for roughly ‘cylindrical’ PTV in thorax and pelvic regions. Beams were placed approximately 70 degrees apart, at gantry angles of 225, 325, 180, 105 and 35 degrees. For head and neck region, we had used seven equally spaced non-opposing beams, and they were placed approximately 50 degrees apart. In all the cases, the isocenter of the beams was at the geometrical center of PTV and which was ITP point also. The planning was performed by TPS with ITP.
5. Dosimeter: Unidos electrometer with 0.125-cc ionization chamber of thimble type. The chamber outer diameter and length is 6.9 and 18.7 mm, respectively.
6. IMRT Phantom: Universal IMRT verification phantom of PTW make [Figure 1]. The dimension of IMRT phantom is 300 mm × 300 mm × 70 mm and is made of Polymethyl Methacrylate (PMMA) material. The measuring depth of ionization chambers is 60 mm.

At first, CT images of the Universal IMRT phantom with 0.125-cc chamber were taken with 2-mm slice thickness. The chamber was put in the central slot provided in the phantom. The CT images were exported to Oncentra virtual simulation software for contouring. The contoured images were imported into Plato 3D TPS for planning. A phantom plan was created for each patient treated with IMRT by superimposing the patient plan on to the IMRT phantom. All gantry angles were made to zero-degree orientation for the measurement without changing anything further so that isodose and profile remained the same, and it was called phantom plan. The dose was calculated at the reference point (ITP point) of the chamber. The reference point of chamber was at the central axis of the beam and at a depth of 6 cm (in the Universal IMRT phantom), i.e., at the isocenter. The isocenter is nothing but ITP of the patient plan. Beam incidence was perpendicular to the flat surface of the phantom. Ionization chambers were oriented with their longitudinal axes perpendicular to the direction of the MLC leaf motion. The dose was measured on PTW Unidose electrometer.

The results were expressed as percentage difference between calculated and measured dose as follows: $\%\text{ Difference}=\frac{\text{Measured dose - Calculated dose}}{\text{Calculated dose}}\times 100$

The results were expressed under a tolerance level of ±3%.

The plan which failed in the tolerance criteria between the measured and calculated dose proceeded in the following way:

1. Beam profiles were generated by the TPS in the GT and cross plane direction in the isocenter plane of the phantom. GT is the ‘Gun to Target’ direction in the LINAC.
2. Marked nine points (including center of IMRT phantom), equidistant (distance between two points was 10 mm) to each other, around the chamber along GT and cross plane in the phantom as shown in Figure 2.

**Figure 2:**
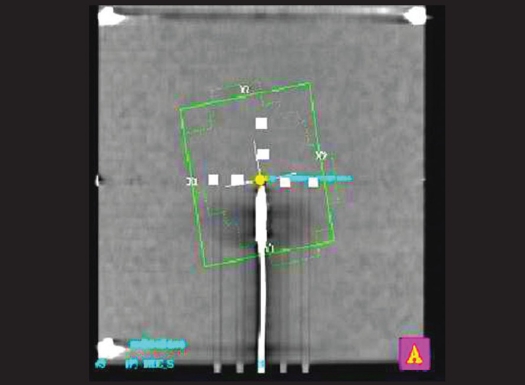
Position of nine different points around the chamber
3. In order to find out the low-dose gradient, the Phantom Plan was generated for each of the marked points at 6-cm depth of the phantom, i.e., the superimposition of the ITP point over these points one by one.
4. The best plan, where the low-dose gradient was found, was exported for measurement.
5. According to the coordinates generated in the TPS, the couch was manually displaced at the required position and point dose was measured. The measured dose was compared with the dose calculated from TPS [Table 1].

**Table 1:** Data for 20 case studies

*Case*	*No. of. fields*	*Deviation from TPS vs point dose measurement (%)*	*Displacement of the couch*	*Deviation from TPS vs point dose measurement after optimization (%)*
Ca Pharynx	17	8	*X* = −2.2 cm, *Y* = 0.3 cm	−0.3
Ca Leiomyosarcoma	7	4.01	*X* = 0.0 cm, *Y* = 0.5 cm	1.0
Ca Larynx	7	5.9	*X* = −1.3 cm, *Y* = 0.2 cm	2.6
Ca Maxilla	7	−5.5	*X* = 2.8 cm, *Y* = 0.1 cm	0.52
Ca Prostate	5	−6.9	*X* = −1.6 cm, *Y* = 0.1 cm	4.0
Ca Endometrium	5	−6.23	*X* = −0.7 cm, *Y* = 0.2 cm	1.1
Ca Esophagus	5	−9.5	*X* = −0.5 cm, *Y* = 0.2 cm	6.0
Ca Thyroid	7	9.89	*X* = 1.1 cm, *Y* = 0.5 cm	1.7
NHL	5	−5.62	*X* = 0.2 cm, *Y* = 1.0 cm	2.3
Ca Stomach	5	10.2	*X* = 1.6 cm, *Y* = 2.0 cm	2.2
Ca Supraglottic larynx	5	11.12	*X*= 0.6 cm, *Y* = 1.1 cm	−3.0
Ca GBM	7	10.52	*X* = −0.7 cm, *Y* = −1.5 cm	6.9
Ca Stomach	5	8.75	*X* = 1.4 cm, *Y* = 0.8 cm	2.4
Ca Prostate	5	−11.41	*X* = 0.8 cm, *Y* = 1.3 cm	5.0
Ca Tongue	7	−6.73	*X* = −0.5 cm, *Y* = 2.1 cm	−1.4
Ca Rectosigmoid Junction	5	5.25	*X* = 1.4 cm, *Y* = 1.3 cm	2.4
Ca Bladder	5	−5.75	*X* = 0.8 cm, *Y* = −1.6 cm	0.58
Ca Gall bladder	5	10.9	*X* = 0.0 cm, *Y* = 1.8 cm	2.9
Ca Stomach	5	−5.72	*X* = −1.0 cm, *Y* = −1.0 cm	2.9
Ca Cx	5	6.78	*X* = 1.6 cm, *Y* = 0.4 cm	2.2

Taking the example of a Ca Pharynx patient plan consisting of seven fields [Table 1], beam profiles were generated in the TPS at the isocenter plane both in the GT and cross plane and are shown in Figures 3a and 3b. In order to find optimized position for point dose measurement, nine different points were taken in the chamber plane as shown in Figure 2. Optimized point dose values were generated in the TPS as shown in Table 2. The couch was manually displaced in the GT and cross plane direction in accordance with the coordinates of the points generated in the TPS. The best point was the point where there was an acceptable dose difference between measured and calculated dose.

**Figure 3a:**
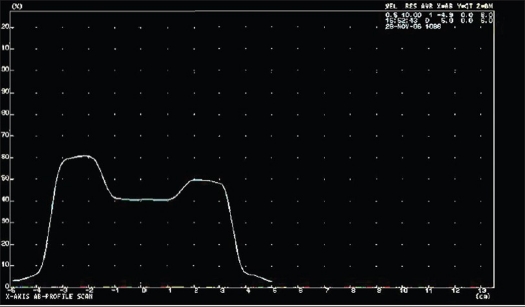
Cross plane of GT axis profile for beam 1

**Figure 3b:**
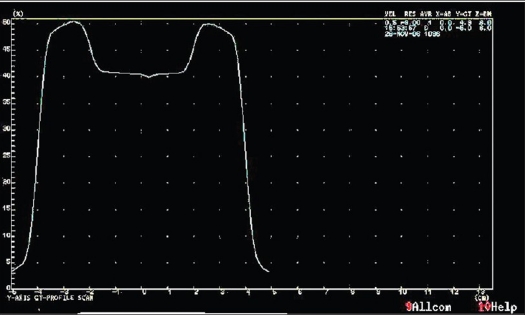
GT axis profile for beam 1

**Table 2:** Optimized point dose values from treatment planning system for ca Pharynx patient plan

*X (cm)*	*Y (cm)*	*Z (cm)*	*Total dose*	*BEAM #1*	*BEAM #2*	*BEAM #3*	*BEAM #4*	*BEAM #5*
1.8	0.2	0.0	263.1	51.7	60.6	40.4	51.8	58.6
3.9	0.3	0.0	28.8	7.7	3.4	4.4	7.3	5.9
−2.2	0.3	0.0	261.7	63.2	47.9	54.8	39.1	56.6
−4.1	0.3	0.0	26.5	6.5	6.2	6.4	4.4	3.1
−0.2	0.3	0.3	263.0	42.4	52.5	55.6	51.7	60.7
2.0	0.3	0.3	260.7	52.2	55.7	43.3	51.7	57.9
3.9	0.2	0.3	31.5	8.5	3.5	4.5	8.1	6.9
−2.4	0.2	0.3	255.0	63.6	47.6	54.0	39	50.7
−4.2	0.3	0.3	27.6	6.3	7.3	6.7	4.3	3.0

## Results and Discussion

We have treated 132 IMRT patients so far. Among them, there were 17 prostate, 17 cervix and endometrium, 32 brain tumors; and rest of them belonged to other extremities. The total number of fields for each treatment plan was 5-7, and the dose per fraction varied between 1.8 and 2 Gy. The breakup of different cases and variation in point dose measurement are shown in Table 3.

**Table 3:** Variation in point dose measurement in various regions (132 patients' plan)

*Brain, head and neck (55) (Astrocytoma, glioma, maxilla, etc.)*		*Thorax (10) (Stomach, esophagus, gall bladder, etc.)*		*Pelvis (48) (Prostate, cervix, endometrium, etc.)*		*Extremities (19) (Sarcoma, myosarcoma, NHL, etc.)*	
			
*No. of cases*	*% Variation between TPS vs measured*	*No. of cases*	*% Variation between TPS vs measured*	*No. of cases*	*% Variation between TPS vs measured*	*No. of cases*	*% Variation between TPS vs measured*
47	Within ±3%	7	Within ±3%	37	Within ±3%	15	Within ±3%
8	Beyond ±3%	5	Beyond ±3%	9	Beyond ±3%	4	Beyond ±3%

The mean percentage deviation value for the 20 cases studied, under 95% confidence interval, was found to be 2.10% ± 1.14.

It has been found that even after optimization, four cases showed more than ±3% deviation [Table 1]. Among those four cases, one was having single solid tumor and others had only nodes to be treated.

The deviation in some fields occurs because of highly inhomogeneous fields, hence absolute dose measurement for IMRT beamlets is difficult due to the lack of lateral electron equilibrium. That is why ionization chambers should typically be placed in low-dose gradient regions.[7] Chamber type and dimension are very important, and smaller volumes are more sensitive to position and will have a higher response when positioned at an opposing leaf pair junction and between adjacent leaves.

**Figure 4:**
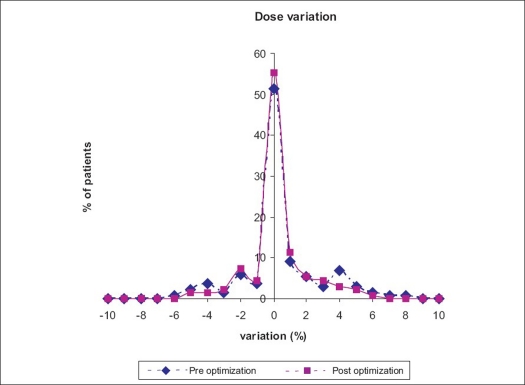
Pre- and post-optimized point dose measurement for 132 IMRT treatment plans

To minimize the effect of volume averaging, the detector should be smaller than the homogenous region of dose to be measured. The ‘Tongue and Groove’ design of adjacent leaves can result in small regions of a field being blocked and therefore having reduced dose. The pre- and post-optimized percentage variation in point dose measurement for 132 IMRT treatment plans is given in Figure 4. The necessary resolution of the detector depends on the resolution of the beamlet grid that is used for planning and sequencing fields for delivery. Partly due to the fact that the planning system calculation algorithm often cannot model transmission, leakage and scattering dose accurately in the low-dose regions; and partly in finding a uniform dose area for that region, the ionization chamber measurements in low-dose region often showed higher dose than predicted by the planning system. Measurements of profile and depth dose curves require stepping up of ion chamber across the field or up the field. This necessitates the displacement of couch, and so the chamber across the field for optimized dose measurement in this study. The generation of optimization point by taking random points is a time-consuming process and needs to be found through a computer algorithm.[8]

## Conclusion

Of the 20 cases studied, 16 were found to be within acceptable criteria after necessary displacement of the couch. For those cases in which percentage variation between TPS-planned dose and measured dose was found to be within unacceptable criteria (beyond ±3% interval) even after optimization, plans were further studied with Film Dosimetry using radiochromic films. The parameters include gamma map, dose difference, distance-to-agreement, etc.; and final ‘accept/reject’ criteria depend upon outcome of these factors.[8]

Hence, point dose measurement at the reference point in the phantom can be an effective tool for patient-specific IMRT verification and QA, which should be further verified with film dosimetry or suitable dosimetry system like portal imaging and gel dosimetry, etc.
